# Label-Free Electrochemical Immunoassay for C-Reactive Protein

**DOI:** 10.3390/bios8020034

**Published:** 2018-03-30

**Authors:** Madasamy Thangamuthu, Christian Santschi, Olivier J. F. Martin

**Affiliations:** Nanophotonics and Metrology Laboratory (NAM), Swiss Federal Institute of Technology Lausanne (EPFL), 1015 Lausanne, Switzerland; christian.santschi@epfl.ch (C.S.); olivier.martin@epfl.ch (O.J.F.M.)

**Keywords:** screen printed electrode (SPE), C-reactive protein, electrochemical assay, label-free Immunosensor, gold nanoparticles

## Abstract

C-reactive protein (CRP) is one of the most expressed proteins in blood during acute phase inflammation, and its minute level increase has also been recognized for the clinical diagnosis of cardio vascular diseases. Unfortunately, the available commercial immunoassays are labour intensive, require large sample volumes, and have practical limitations, such as low stability and high production costs. Hence, we have developed a simple, cost effective, and label-free electrochemical immunoassay for the measurement of CRP in a drop of serum sample using an immunosensor strip made up of a screen printed carbon electrode (SPE) modified with anti-CRP functionalized gold nanoparticles (AuNPs). The measurement relies on the decrease of the oxidation current of the redox indicator Fe^3+^/Fe^2+^, resulting from the immunoreaction between CRP and anti-CRP. Under optimal conditions, the present immunoassay measures CRP in a linear range from 0.4–200 nM (0.047–23.6 µg mL^−1^), with a detection limit of 0.15 nM (17 ng mL^−1^, S/N = 3) and sensitivity of 90.7 nA nM^−1^, in addition to a good reproducibility and storage stability. The analytical applicability of the presented immunoassay is verified by CRP measurements in human blood serum samples. This work provides the basis for a low-priced, safe, and easy-to-use point-of-care immunosensor assay to measure CRP at clinically relevant concentrations.

## 1. Introduction

To advance cardiovascular risk assessment, attention has focused on monitoring C-reactive protein (CRP), which is a biomarker for inflammation. It has been shown in multiple studies that incident myocardial infarction, stroke, atherosclerosis, peripheral arterial disease, and sudden cardiac death are associated with CRP [1]. Indeed, several clinical studies have shown that an elevated level of CRP correlates well with cardio vascular risk; i.e., concentrations that are lower than 1 mg L^−1^ indicates low risk, 1–3 mg L^−1^ moderate risk, and 3–10 mg L^−1^ high risk [2,3,4]. Hence, the measurement of CRP has become important for early risk assessment of cardiac diseases and taking appropriate measures to reduce the number of cardiovascular related deaths. 

In routine clinical laboratories, serum CRP is usually estimated by turbidimetric [5], microplate reader [6], nephelometric technologies [7], and enzyme-linked immunosorbent assay (ELISA) that are commercially available kits (Cell Biolabs, Inc. San Diego, CA, USA, catalog no. STA-392; Thermofisher Scientific, Vienna, Austria, catalog no. KHA0031). These assays are well established, reproducible, and show sufficient sensitivity. However, they are not appropriate for point-of-care analysis as these techniques are time-consuming (require highly-skilled personnel), highly expensive, susceptible to false negatives, and are only available in well-equipped laboratories. To overcome these limitations, CRP quantification using surface plasmon resonance (SPR) [8,9], piezoelectric microcantilevers [10], quartz crystal microbalance technology [11], microfluidics [12], and electrochemical methods [13,14] have been developed. Especially, electrochemical immunoassays are most promising in terms of high sensitivity, lower detection limit, fast response, low-cost, ease of handling and miniaturization. Although extensive research efforts have been carried out, the development of a simple and highly performant CRP immunoassay is still challenging for point-of-care analysis. 

Capacitive electrochemical immunoassays are potential candidates for hand-held point-of-care devices [15,16]. However, the non-specificity and large background signals are limiting this type of sensors, and, therefore, researchers are seeking more specific and sensitive label-free electrochemical immunosensors for CRP. For such a configuration, external redox mediator ferrocyanide, Fe(CN)_6_^4−^ is highly desirable to produce a change in the electrochemical current response, which is controlled by the immunoreaction between the CRP and anti-CRP [17]. Furthermore, it does not require secondary antibody and labeling, thus making fabrication very simple, less expensive, immune to the influence from conjugated markers of different agents, and efficient for the antigen-antibody binding. Earlier reports that were published on label-free CRP measurements [13,17,18] are limited in sensitivity, and do not encompass a clinically relevant range. Subsequently, to promote the sensitivity and the stability of the electrodes, nanomaterials, such as carbon nanotubes, gold nanoparticles (AuNPs), quantum dots, graphene, and metal oxide nanoparticles have been in the focus of interest [19,20,21,22,23,24]. In this context, Zhu et al. reported the AuNPs amplified label-free electrochemical immunosensor for CRP [25]. Bryan et al. used polycrystalline gold to enhance the anti-CRP immobilization and obtained a sensitive electrochemical impedimetric CRP sensor [26]. Recently, Gupta et al. reported a sensitive CRP immunosensor using carbon nanofibers [27]. Although these sensors have sufficient sensitivity, fabrication seems to be quite complex and CRP measurements in a drop of sample remain challenging. 

Nowadays, screen printed electrodes (SPE) are most promising to reduce the sample volume and to fabricate a simple, low-cost electrochemical immunosensor; they are robust transducers that permit miniaturization and enable the integration of the reference and working electrodes in the same chip. There are a few sandwich CRP immunoassays that are reported using SPE [28,29,30], but, to the best of our knowledge, there is no report on label-free electrochemical immunoassay for CRP using SPE. Therefore, for the first time, we have developed a miniaturized, low-cost, label-free electrochemical immunoassay for CRP using SPE that is modified with anti-CRP functionalized gold nanoparticles (AuNPs). The measurement relies on monitoring the decrease in the oxidation current of the Fe(CN)_6_^4−^, resulting in the formation of irreversible immunocomplex on the immunosensor surface. The alteration of SPE with AuNPs provides an appropriate matrix which is adapted to the immobilization of anti-CRP, and acts, moreover, as an electron communicator between the Fe^3+^/Fe^2+^ redox complex and the SPE. Low detection limit, high sensitivity, large detection range, excellent repeatability, and stability underline the solid performance of the presented immunoassay. The analytical applicability of the present immunoassay is validated for blood serum samples.

## 2. Materials and Methods

### 2.1. Reagents and Chemicals

Human blood serum (male AB plasma USA origin), human CRP, anti-human CRP developed in goat, bovine serum albumin (BSA), potassium ferrocyanide K_4_[Fe(CN)_6_], L-cysteine, potassium chloride (KCl), potassium nitrate (KNO_3_), chloroauric acid trihydrate (HAuCl_4_·3H_2_O), and 1-Ethyl-3-(3-dimethylaminopropyl) carbodiimide (EDC), *N*-hydroxy succinamide (NHS) were purchased from Sigma-Aldrich (Buchs, Switzerland). Milli-Q water and 0.1 M PBS (pH 7.2) were used for reagent preparation and dilution. The PBS was deaerated prior to use.

### 2.2. CRP Measurements in Blood Serum

The blood serum was diluted 10 times in PBS. A 30 µL of the sample (or CRP standard solution) was placed onto the imunosensor strips and the concentration of CRP was measured using amperometric technique. After an incubation time of 30 min, the electrodes were washed with PBS and a drop of 50 µL of 0.5 mM ferrocyanide that was prepared in PBS was dropped onto the electrodes. Finally, a standard addition calibration curve was used to quantify the concentration of CRP. Throughout, the dilution factor was taken into account. 

### 2.3. Instrumentation

The three electrode electrochemical workstation CHI 1240B (CH Instruments, Austin, TX, USA) was used for cyclic voltammetric and amperometric studies. Screen printed electrode (TE100, CH Instruments, Austin, TX, USA) comprising of carbon counter electrode, an Ag/AgCl reference electrode, and anti-CRP functionalized AuNPs modified working electrode were used as immunosensor strip. The working electrode surface area was 0.071 cm^2^. Once the preparation was accomplished, the immunosensor strip was equilibrated in PBS cycling the voltammetric potential between −0.6 V and +0.6 V until the current stabilised. A scanning electron microscope (SEM) (Carl Zeiss, Jena, Germany) with an electron energy adjusted to 2 keV was used to obtain the morphological images of the samples. 

### 2.4. Immunosensor Fabrication

**Figure 1:**
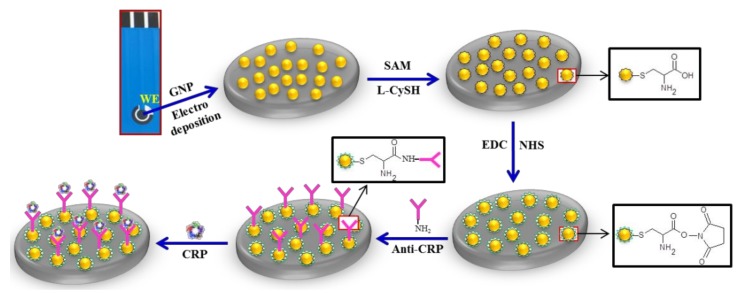
Schematic representation of the fabrication steps for the label-free C-reactive protein (CRP) immunosensor.

Prior to modifying the immunosensor, the SPE was pre-treated in 1 M H_2_SO_4_ solution. A potential ranging from −0.5 to +1.0 V was swept for 10 cycles at a scan rate of 50 mV s^−^^1^. Such a treatment removes the organic ink constituents/contaminants and increases the surface functionalities. Then, the AuNPs were electrodeposited by placing the mixture of 0.5 mM HAuCl_4_·3H_2_O and 0.1 M KNO_3_ solution onto the SPE and sweeping the potential from +0.9 to 0 V for five cycles at a rate of 50 mV s^−^^1^. To functionalize the AuNPs with anti-CRP, a self-assembled monolayer (SAM) of cysteine with a carboxylic acid (-COOH) functional head group was anchored by its AuNPs-thiol bond. Subsequently, 1 mM L-cysteine solution was poured onto the AuNPs-SPE and kept at room temperature for 4 h. After the SAM was formed, the surface of the working electrode was washed with ethanol and water before drying with a flow of nitrogen gas. In order to ensure a stable terminal activation of -COOH groups, the SAM was treated with coupling reagents containing 0.4 M EDC and 0.1 M NHS for 30 min prior to gentle rinsing cycles using PBS twice to remove the poorly attached linker molecules. Subsequently, the prepared surface was incubated with a 10 µM anti-CRP solution at an ambient temperature for 1 h. The reaction of the primary amine (-NH_2_) groups of the antibodies with the -COOH groups on the AuNPs resulted in a formation of stable amide bonds, as schematically shown in Figure 1. The loosely-bonded anti-CRP was removed from the electrode by rinsing with PBS before the anti-CRP-SAM-AuNPs-SPE was immersed in a 5 wt% bovine serum albumin (BSA) solution at room temperature for 1 h to block the remaining non-specific binding of antibody active sites. Finally, the ready-to-use surfaces were stored at 4 °C.

### 2.5. Immunoreaction of CRP with Anti-CRP

For evaluating the immunoreaction between CRP and anti-CRP, a known concentration of CRP that was prepared with PBS solution was placed over the sensing element and was allowed to react with anti-CRP for 30 min. To remove the non-specifically attached CRP, the electrode was thoroughly washed with PBS and dried before every electrochemical experiment.

## 3. Results and Discussion

### 3.1. Morphological Characterization

**Figure 2:**
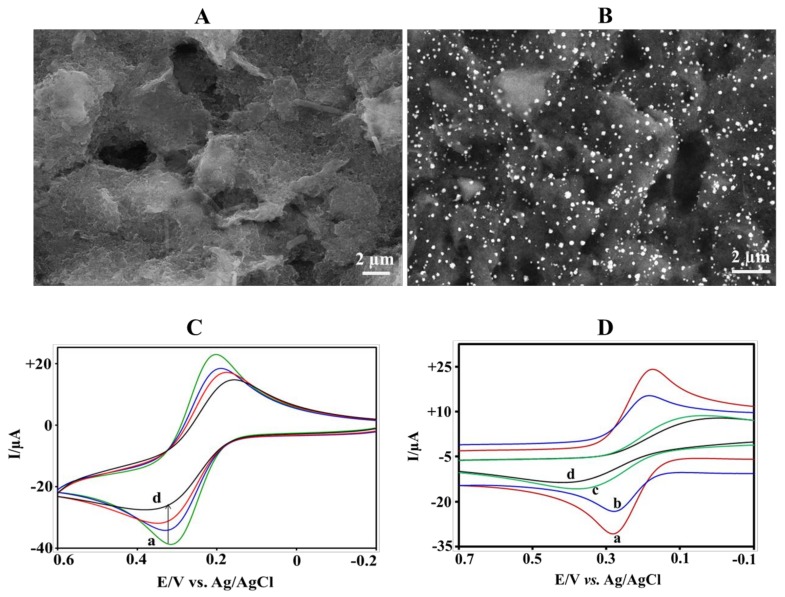
Scanning electron microscope (SEM) images of (**A**) bare screen printed electrodes (SPE) and (**B**) AuNPs-SPE. (**C**) Electrochemical response for (a) AuNPs-SPE, (b) anti-CRP-self-assembled monolayer (SAM)-AuNPs-SPE, (c) BSA-anti-CRP-SAM-AuNPs-SPE and (d) 1 nM CRP-anti-CRP-SAM-AuNPs-SPE in PBS solution containing 0.5 mM Fe(CN)_6_^4−^. (**D**) Typical cyclic voltammetric responses of the AuNPs modified electrode (a) SAM-AuNPs-SPE and (b) anti-CRP-SAM-AuNPs-SPE, and without AuNPs electrode (c) bare SPE, and (d) anti-CRP-SPE for 1 nM CRP in PBS containing 0.5 mM Fe(CN)_6_^4−^ at a scan rate of 50 mV s^−1^.

The morphological images of the bare SPE and AuNPs modified SPE are shown in Figure 2A,B. The SEM images clearly reveal a porous surface of the bare SPE. Generally, using cyclic voltammetry electrodeposition, ≈20–60 nm nanoparticles are randomly deposited over the whole conducting electrodes as observed in our study. Such a configuration is suited for the immobilization of anti-CRP without impairing its biological activity, and, moreover, facilitates electron transfer between SPE and electrochemical probe.

### 3.2. Electrochemical Characterization

The electrochemical characteristics of the AuNPs-SPE, anti-CRP-SAM-AuNPs-SPE, BSA-anti-CRP-SAM-AuNPs-SPE, and CRP-anti-CRP-SAM-AuNPs-SPE (1 nM CRP) were investigated using cyclic voltammetry in PBS containing 0.5 mM Fe(CN)_6_^4−^ at a scan rate of 50 mV s^−^^1^. Typical electrochemical responses are shown in Figure 2C. The highest peak current was obtained for the AuNPs-SPE (curve a) and a decreased current response was observed after the anchoring of BSA and immobilization of anti-CRP (curve b and c). The successful immobilization of anti-CRP on the surface of SAM-AuNPs-SPE leading to the formation of electron blocking layers which hinders the electron transfer efficiently. The current response is decreased after incubation in 1 nM CRP (curve d). This can be attributed to the insulating protein layer that is formed on the electrode, which further hinders the electron transfer.

To evidence the effect of AuNPs, bare SPE, SAM-AuNPs-SPE, anti-CRP-SAM-AuNPs-SPE, and anti-CRP-SPE were incubated in 1 nM CRP and their electrochemical responses were recorded by applying the same conditions as described above. The corresponding responses are shown in Figure 2D. It has been observed that in the absence of the antibodies, SAM-AuNPs-SPE (curve a) exhibits a higher redox current than bare SPE (curve c), because AuNPs provide higher surface area and enhanced electron transfer rates. In the presence of antibodies, both anti-CRP-SAM-AuNPs-SPE (curve b) and anti-CRP-SPE (curve d) show reduced currents when compared to their correspoding counterparts. The current in the AuNPs modified SPE is reduced by 13 μA (from 33 μA to 20 μA), whereas the bare SPE shows only a 3 μA current reduction (from 16 μA to 13 μA). The more pronounced current reduction on anti-CRP-SAM-AuNPs-SPE can be attributed to the larger amounts of immobilized antibodies, which is confirmed by the estimated surface coverage values using the Randles-Sevcik’s equation [31,32].

(1) Ip = (2.69 ×105)An32D12Cγ12,

where, *C* and *D* correspond to the concentration and diffusion coefficient of Fe(CN)_6_^4−^, respectively. The parameters *I_p_*, *n*, *γ,* and *A* are attributed to the maximum current response, the number of transferred electrons per molecule, the scan rate, and the effective surface area. The calculated surface coverage of anti-CRP on SAM-AuNPs-SPE was 22.6 nmol cm^−2^, which is about 13 times higher than for the bare SPE (1.7 nmol cm^−2^), suggesting that the enhanced immobilization of anti-CRP is related to the larger surface area and the higher electron transfer rate that is offered by the spherically shaped AuNPs (20–60 nm) [31].

**Figure 3:**
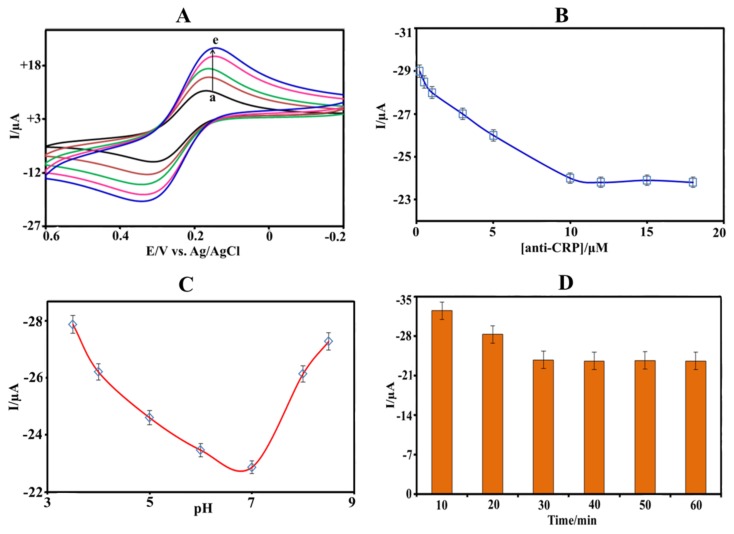
(**A**) Influence of the scan rate on the current response of the label free CRP immunosensor exposed to 1 nM CRP. The scan rate is increased by 10 mV s^−1^ from (a) 10 mV s^−1^ to (e) 50 mV s^−1^. (**B**) Effect of anti-CRP concentration on the cyclic voltammetric response of the SAM-AuNPs-SPE. (**C**) Effect of pH value (scan rate: 50 mV s^−1^). (**D**) Effect of incubation time on the current responses of the CRP immunosensor to 1 nM CRP.

The optimized gold salt concentration (0.5 mM) and electrodeposition time (180 s, i.e., five cycles) were used from our earlier report [33] to achieve a maximum electron transfer property of the AuNPs. To investigate the characteristics of the surface reaction, cyclic voltammograms for various scan rates ranging from 10 to 50 mV s^−^^1^ were recorded, as displayed in Figure 3A. The resulting current responses linearly increased with the square root of the scan rate, suggesting that the reaction is diffusion-controlled [34]. 

### 3.3. Optimization

The performance of the immunosensor is mainly influenced by the antibody concentration, pH, and CRP incubation time, and, hence, these parameters have been carefully optimized. The electrochemical response of the SAM-AuNPs-SPE decreases while increasing the anti-CRP concentration from 200 nM to 10 μM and stabilizes at higher concentration, as shown in Figure 3B. The electrode was saturated with anti-CRP above 10 μM since further increasing the concentration did not influence the current response. Therefore, 10 μM anti-CRP concentration was used for the fabrication of the immunosensors. The electrochemical activity of the CRP immunosensor, incubated under the same conditions as described above, was investigated in the pH range from 3.5 to 8.5 as shown in Figure 3C. At constant oxidation peak potential (+0.3 V) of the electrochemical probe, more reduced current was observed at pH 7.0 indicating that more anti-CRP is present on the surface. Upon increasing and decreasing the pH from 7, less reduced current was observed, suggesting that the biomolecules CRP and anti-CRP are not stable at highly acidic or alkaline conditions where they lose their activity. Hence, for further measurements, pH 7.0 was selected as it appears to be the optimal pH value for CRP measurements. 

The effect of incubation time on the amperometric current response was furthermore investigated. During incubation, the CRP reaches the anti-CRP on the SAM-AuNPs-SPE surface and takes some time to form an immunocomplex. With an increasing incubation time, the current response decreases and stabilizes after 30 min, as shown in Figure 3D. It indicates that the capacity of the CRP to form immunocomplex with anti-CRP on the immunosensor strip gradually tends to saturation. It turns out that an incubation time of 30 min is found to be sufficient to obtain an optimal current response.

### 3.4. CRP Measurement

**Figure 4:**
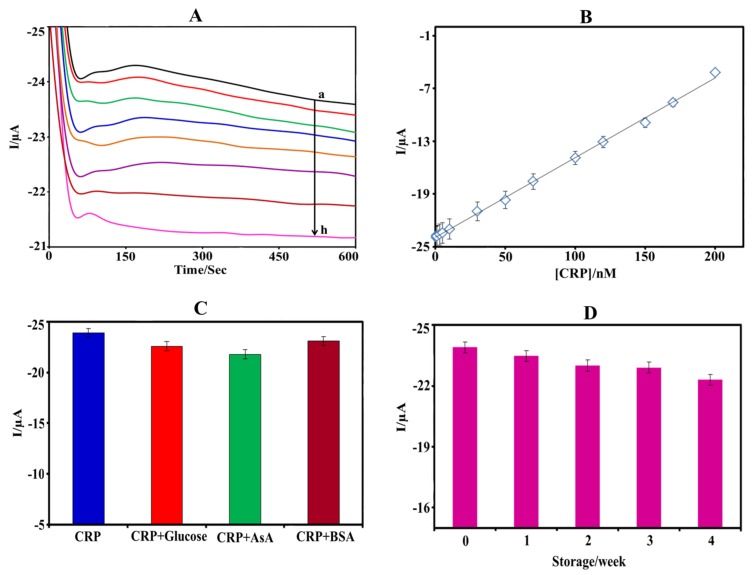
(**A**) Time vs. current response of the CRP immunosensor for (a) 1, (b) 2, (c) 5, (d) 7, (e) 10, (f) 15, (g) 20, and (h) 25 nM of CRP. (**B**) Calibration chart of the anodic oxidation current vs. CRP concentration ranging from 0.4–200 nM (*y* = 0.09*x* ‒ 23.9, *r*^2^ = 0.9985). The values for each point were averaged over three measurements. (**C**) Histograms representing the selectivity of the label-free CRP immunosensor toward CRP, CRP + Glucose, CRP + ascorbic acid, and CRP + BSA in PBS solution containing 0.5 mM Fe(CN)_6_^4−^ at a scan rate, 50 mV s^−1^. (**D**) Histograms representing the stability of the immunosensor strip under the same working conditions as described in the text.

Under optimized conditions, the determination of CRP concentration was performed using amperometry technique at constant oxidation potential +0.3 V. The electrochemical response of the anti-CRP-SAM-AuNPs-SPE was considered as base line (control) and the corresponding variations with respect to the concentration of the CRP were measured. Figure 4A shows the amperometric (time vs. current) current responses of the present label-free CRP immunosensor for various concentrations of CRP. It can clearly be seen that the current signal decreases gradually with an increasing CRP concentration since the number of bound CRP increases, forming immunocomplexes that block the electron transfer of the probe, Fe(CN)_6_^4−^. The observed oxidation currents, measured after a time lapse of 500 s, vs. the CRP concentration are plotted in Figure 4B. The resulting calibration unveiled a linear range from 0.4–200 nM CRP (0.047–23.6 µg mL^−1^) with a detection limit of 0.15 nM (17 ng mL^−1^, S/N = 3) and a sensitivity of 90.7 nA nM^−1^. The electroanalytical characteristics (detection limit, linear range, and sensitivity) of the immunosensor are similar to earlier that reported label-free methods as shown in Table 1. Although, earlier reported immunosensors show better detection limit, the detection limit that is achieved using our immunosensor strips is sufficient enough to measure CRP in physiological samples. Furthermore, the presented label-free electrochemical immunosensor assay is more suitable for point-of-care applications as it can measure CRP in a drop of sample using low-cost disposable strip when compared to the earlier reported immunosensors. Hence, the presented CRP immunosensor is highly suitable for the early detection of cardiovascular diseases.

### 3.5. Selectivity, Stability, and Reproducibility

Since the determination of CRP concentration is based on the selective immunoreaction between CRP and anti-CRP, the present immunosensor is highly selective [35]. However, some possible interfering substrates that potentially co-exist in human serum, such as glucose, ascorbic acid, and BSA, were investigated to evaluate the specificity and are shown in Figure 4C. The immunosensor strip was incubated with 2 nM of CRP in the presence of 10 nM interfering agents. The observed current responses vary less than 10% (Table 2, each analyte is normalized to CRP corresponding to 100%), even when the concentration of the interfering substance is five times higher than the CRP concentration, indicating an excellent specificity of the present label-free electrochemical immunoassay.

The stability of the immunosensor was tested when stored at 4 °C. The electrochemical current responses decreased by 3.6% after two weeks, and 6.8% after four weeks (Figure 4D), indicating that the activity of the antibody is retained. The evaluation of the immunosensors reproducibility is essential for the reliability of the present immunoassay. It was investigated by fabricating four independent immunosensor strips and their current responses varied only by 2.3% (standard deviation), thus confirming that the measurements are highly reproducible. The immunosensor is fabricated on disposable screen printed electrodes, and is, therefore, predestined for one time use.

### 3.6. Real Sample Analysis

There are several amperometric sandwich immunoassays that are reported for point-of-care CRP analysis [14,28,29,30,36,37]. To the best of our knowledge, we are the first reporting a label-free electrochemical CRP immunoassay to measure the CRP concentration in a drop of serum sample using SPEs that are modified with anti-CRP functionalized AuNPs. The applicability of the present immunoassay was validated by estimating the CRP concentration in human serum samples using a standard addition technique. A drop of the serum sample was placed onto the modified SPE and the corresponding amperometric current responses were recorded. The CRP concentration was quantified for each addition, as shown in Table 3, by converting the current into concentration using the calibration curve. Each value represents the average of three measurements. The accuracy of the present immunoassay was tested by determining the retrieval of the known amounts of CRP that was added to the serum samples. The retrieved values between 93.6% and 103.2% of the known amounts indicate a good accuracy. The CRP concentration in the examined serum sample was found to be 7.9 nM or 0.932 mg/L, indicating that the commercial serum was withdrawn from humans with a clinically reported normal range [2]. Further, the dynamic linear range of the present immunoassay covers from normal to extreme levels of CRP concentration in blood, and, hence, the present immunoassay can potentially be used for point-of-care CRP measurements.

## 4. Conclusions

In summary, a simple label-free electrochemical immunoassay has been successfully tested for the measurement of the important cardiac biomarker CRP using SPE modified with anti-CRP functionalized AuNPs. The well characterized and optimized immunosensor strip shows good analytical performances, such as wide linear detection range, low detection limit, and high sensitivity, in addition to excellent reproducibility and stability. The present immunoassay enabled for measuring CRP concentrations in human blood serum samples, and is, hence, highly suited for quality health care management. It could be adopted for point-of-care analysis. Furthermore, the presented configuration could inspire researchers to develop further label-free electrochemical immunosensors for the measurement of different clinical biomarkers. 

## Figures and Tables

**Table 1:** Electroanalytical properties of earlier reported label free CRP immunosensors, including the present work.

Immunosensor	Linearity (nM)	Sensitivity (nA nM^−1^ cm^−2^)	Detection Limit (nM)	Reference
antiCRP-SAM-Au	0.5–50	-	0.176	[26]
antiCRP-VACNFs-SiO_2_	0.42–42	-	0.090	[27]
antiCRP-SAM-Au	0.097–9.74	-	0.050	[13]
antiCRP-SWCNT-FET	0.003–0.847	-	0.003	[18]
antiCRP-SAM-AuNPs-SPE	0.4–200	90.7	0.150	Present work

SAM-Self-assembled monolayer; VACNFs—Vertically aligned carbon nanofibers; SiO_2_—Silicon dioxide; SWCNT-Single walled carbon nanotubes; FET-Field effect transistor.

**Table 2:** Selectivity of the present CRP immunosensor strip.

Analyte	Selectivity (%)
CRP	100
CRP + Glucose	93
CRP + Ascorbic acid	91
CRP + Bovine serum albumin	96

**Table 3:** Measurements of CRP antigen concentrations in human blood serum samples.

Sample No.	CRP Added (nM)	CRP Measured (nM)	Recovery (%)	RSD (%)
1	5	5.05	101.2	1.9
2	10	9.36	93.60	2.3
3	15	14.14	94.30	1.4
4	20	20.64	103.2	2.7
5	25	24.45	97.80	1.8
